# Application, knowledge and training needs regarding comprehensive geriatric assessment among geriatric practitioners in healthcare institutions: a cross-sectional study

**DOI:** 10.1186/s12877-024-04964-9

**Published:** 2024-04-18

**Authors:** Shanshan Shen, Xingkun Zeng, Xiaoliang Hui, Lingyan Chen, Jinmei Zhang, Xujiao Chen

**Affiliations:** 1 Department of Geriatrics, Zhejiang Hospital, No. 12 Lingyin Road, Hangzhou, 310013 Zhejiang Province P.R. China; 2 Department of Nursing, Zhejiang Hospital, Hangzhou, China

**Keywords:** Application, Knowledge, Training need, Comprehensive geriatric assessment, Geriatric syndrome

## Abstract

**Background:**

This study aimed to investigate the actual application, knowledge, and training needs of comprehensive geriatric assessment (CGA) among geriatric practitioners in China.

**Methods:**

A total of 225 geriatric practitioners attending the geriatric medicine or geriatric nursing training were recruited for this cross-sectional study. The questionnaire included demographics, healthcare institution characteristics, the actual application, knowledge, training needs, and barriers to CGA and geriatric syndromes (GS).

**Results:**

Physicians and nurses were 57.3% and 42.7%, respectively. 71.1% were female, with a median age was 35 years. Almost two-thirds (140/225) of geriatric practitioners reported exposure to CGA in their clinical practice. The top five CGA evaluation items currently used were malnutrition risk (49.8%), fall risk (49.8%), activity of daily living (48.0%), pain (44.4%), and cognitive function (42.7%). Median knowledge scores for the management procedures of GS ranged from 2 to 6. Physicians identified medical insurance payment issues (29.5%) and a lack of systematic specialist knowledge and technology (21.7%) as the two biggest barriers to practicing geriatrics. Nurses cited a lack of systematic specialist knowledge and technology (52.1%) as the primary barrier. In addition, physicians and nurses exhibited significant differences in their knowledge of CGA-specific evaluation items and management procedures for GS (all *P* < 0.05). However, there were no significant differences in their training needs, except for polypharmacy.

**Conclusions:**

The rate of CGA application at the individual level, as well as the overall knowledge among geriatric practitioners, was not adequate. Geriatric education and continuous training should be tailored to address the specific roles of physicians and nurses, as well as the practical knowledge reserves, barriers, and training needs they face.

**Supplementary Information:**

The online version contains supplementary material available at 10.1186/s12877-024-04964-9.

## Background

China’s healthcare system faces a major challenge due to its aging population. To provide comprehensive and continuous healthcare services to the elderly, strategic measures for Health China have been implemented. These measures include establishing geriatric departments and age-friendly medical institutions, with a focus on providing comprehensive geriatric assessment (CGA) services. CGA is a multidisciplinary diagnostic and therapeutic process that aims to identify the medical, psychosocial, functional capabilities, and social environment of an older adult. The goal is to formulate and initiate a coordinated and integrated plan for treatment and follow-up [1, 2]. CGA is an effective approach for screening geriatric syndromes (GS), which are a set of complex symptoms that have a high prevalence in older adults, rather than specific disease categories [3]. GS is associated with a significant burden of disease and comorbidity, which can complicate the management of chronic disease and lead to poor clinical outcomes [4–7]. A longitudinal study of the Women’s Health Initiative Observational Study found that 76.3% of the older women had at least one GS, and a higher number of GS was significantly associated with an increased risk of incident disability [8]. The Mayo Ambulatory Geriatric Evaluation study showed that older patients with GS, such as a history of two or more falls, weight loss, and depressed mood, were more likely to require hospitalization or emergency department visits within one year [7]. The application of CGA has resulted in numerous benefits across different healthcare settings and disease management [9–12].

CGA is considered a crucial skill that geriatric medical teams need to master. The primary workforce responsible for implementing CGA in hospital settings are physicians and nurses in geriatric medical teams. Although experts have reached a consensus on the application of CGA to improve the knowledge of CGA and provide the available standard operating procedures for real clinical settings [13], its actual implementation of CGA in China is still relatively limited [14, 15]. Geriatricians in general hospitals typically come from internal medicine backgrounds, and still focus on specialized diseases in disease management. Knowledge of comorbidities and GS is limited, and CGA is not routinely performed [16]. A study found that the knowledge of CGA and GS among geriatric practitioners is uneven [17]. Identification of existing knowledge and survey of training needs can contribute to the development of targeted continuing medical education in geriatrics [18]. This can further promote the application of CGA in clinical practice. This study aimed to investigate the actual application of CGA at the individual level, as well as the knowledge and training needs for geriatric practitioners to implement CGA in the context of population aging in China.

## Methods

### Study design

A cross-sectional study with a convenience sample was carried out between June 2021 and July 2022 before the geriatric medicine or geriatric nursing training. An online questionnaire was conducted to collect data.

### Participants

A total of 225 geriatric practitioners who attended the geriatric medicine or geriatric nursing training were from 164 healthcare institutions of different ranks in Zhejiang Province. Among them, 73 tertiary hospitals were involved. In addition, 129 cases were physicians and 96 were nurses.

This study was approved by the Medical Ethics Committee of Zhejiang Hospital.

### Measurements

Demographic data included age, sex, educational level, professional qualifications, years of practice in geriatric medicine and nursing, and current workplace. Healthcare institution characteristics included location and rank. The questionnaire had five questions (Q1-Q5) designed to reflect the actual application of CGA at the individual level, two questions (Q6-Q7) designed to reflect participants’ knowledge of management procedures for GS and multidisciplinary team management for the elderly, and three questions (Q8-Q10) designed to reflect geriatric-related training needs and barriers, as detailed as Table S1.

Participants who had systematically and piecemeal training experience were classified as having training experience group, otherwise as no training experience group.

### Data collection process

The potential participants were informed about the opportunity to volunteer for the questionnaire. They were then asked to confirm their willingness to participate and complete the questionnaire individually. All questionnaire items were required to be completed upon submission. The questionnaire with logical errors would be eliminated.

### Sample size calculation

A sample size of 192 participants was calculated to detect the assumed percentage of CGA application (p) of 60%, based on a previous study [], assuming a type I error (α) of 0.05, and a desired precision (d) of 0.05 for a two-sided test. N represents the estimated annual cases of 400 potential participants who attended various forms of geriatric medicine or geriatric nursing training. A non-response rate of 15% was assumed, requiring a total of 221 participants. The formula is as follows:

$$n=\frac{\left({\displaystyle\frac{z_\alpha}\delta}\right)^2\ast p\ast\left(1-p\right)}{\displaystyle\frac{1+\left[\left(\frac{z_\alpha}\delta\right)^2\ast p\ast\left(1-p\right)\right]}N}$$

### Statistical analysis

Data were analyzed using SPSS 26.0 software (SPSS, Chicago, IL, USA). Descriptive statistics were presented as median (interquartile range, IQR), and numbers (percentages) based on variables type and data distribution. The Mann-Whitney U tests and the χ2 tests were used to estimate differences between physicians and nurses. A *P*-value of < 0.05 was considered statistical significance.

## Results

### Demographic characteristics

Out of the 230 questionnaires received, 225 were analyzed after excluding 5 due to logical errors. Table 1 displays the demographic characteristics of physicians and nurses. Physicians and nurses were 57.3% and 42.7%, respectively. 71.1% were female, with a median age was 35 years. Among the physicians, 64 (49.6%) were female, with a median age of 40 years. All the nurses in the study were female, with a median age of 32.5 years. More than 70% of the participants held intermediate and senior professional titles. However, fewer than 30% of the participants currently work in a geriatric department, and the median of years of practice in geriatrics was 2.

**Table 1:** Demographic characteristics of physicians and nurses

	Total (*n* = 225)	Physicians (*n* = 129)	Nurses (*n* = 96)	*P* Value
Age, years, median (IQR)	35(9.0)	40(13.0)	33(6.0)	**< 0.001**
Age ≥ 35 years, n (%)	122(54.2)	87(67.4)	35(36.5)	**< 0.001**
Female, n (%)	160(71.1)	64(49.6)	96(100)	**< 0.001**
University degree or above, n (%)	209(92.9)	121(93.8)	88(91.7)	0.538
Professional qualifications, n (%)				**< 0.001**
Senior title	56(24.9)	54(41.8)	2(2.0)	
Intermediate title	104(46.2)	52(40.3)	52(54.2)	
Junior title or below	65(28.9)	23(17.9)	42(43.8)	
Years of practice in geriatric medicine and nursing, years, median (IQR)	2(5.0)	2(5.0)	3(6.0)	0.993
Years of practice in geriatric medicine and nursing ≥ 3 years, n (%)	103(45.8)	54(41.9)	49(51.0)	0.172
Current workplace, n (%)				0.768
Geriatric department	61(27.1)	34(26.4)	27(28.1)	
Non-geriatric department	164(72.9)	95(73.6)	69(71.9)	

*IQR* interquartile range; Significance difference *P* < 0.05 was shown in bold

### CGA actual application at the individual level

Of 225 participants, 140 reported exposure to CGA in their clinical practices. The vast majority (97.9%, 98.6%, and 98.6%, respectively) agreed that CGA contributed to clinical diagnosis and treatment, clinical care, and ward safety. Additionally, 87.1% of participants reported difficulties in implementing CGA in their clinical practice. The CGA evaluation items that were most frequently assessed were malnutrition risk (49.8%), fall risk (49.8%), activity of daily living (48.0%), pain (44.4%), and cognitive function (42.7%). A comparison between nurses and physicians showed that physicians were more likely to evaluate comorbidity, emotions, and frailty, but paid less attention to assessing pain and fall risk (all *P* < 0.05), as shown in Table 2.

**Table 2:** CGA actual application in the individual level

	Total (*n* = 225)	Physicians (*n* = 129)	Nurses (*n* = 96)	*P* Value
Have you had any experience with CGA in your clinical work? N (%)				0.838
Never/unknown	85(37.8)	47(36.4)	38(39.6)	
Rarely/Occasionally	109(48.4)	63(48.8)	46(47.9)	
Often/Always	31(13.8)	19(14.7)	12(12.5)	
CGA specific evaluation items, n (%)				
Comorbidity	68(30.2)	52(40.3)	16(16.7)	**< 0.001**
Medication	76(33.8)	49(38.0)	27(28.1)	0.122
Hearing and vision	51(22.7)	30(23.3)	21(21.9)	0.807
Oral and swallowing functions	90(40.0)	53(41.1)	37(38.5)	0.700
Malnutrition risk	112(49.8)	65(50.4)	47(49.0)	0.832
Delirium	43(19.1)	26(20.2)	17(17.7)	0.644
Cognitive function	96(42.7)	58(45.0)	38(39.6)	0.420
Emotion	75(33.3)	52(40.3)	23(24.0)	**0.010**
Sleep quality	84(37.3)	54(41.9)	30(31.3)	0.104
Pain	100(44.4)	50(38.8)	50(52.1)	**0.047**
Activity of daily living	108(48.0)	59(45.7)	49(51.0)	0.431
Muscle strength	46(20.4)	28(21.7)	18(18.8)	0.587
Frailty	44(19.6)	31(24.0)	13(13.5)	0.050
Physical function	53(23.6)	35(27.1)	18(18.8)	0.143
Fall risk	112(49.8)	55(42.6)	57(59.4)	**0.013**
Urinary incontinence	53(23.6)	32(24.8)	21(21.9)	0.608

*CGA* comprehensive geriatric assessment; Significance difference *P* < 0.05 was shown in bold

### Knowledge toward management procedures for GS and multidisciplinary team management for the elderly

Table 3 shows that over 80% of participants had experience in diagnosing, treating, or caring for GS. The median knowledge scores of the management procedures for GS and multidisciplinary team management for the elderly ranged from 2 to 6. Worse knowledge was observed in areas such as sarcopenia, frailty, delirium, and comorbidity. Significant differences were found in the knowledge of management procedures for comorbidity, polypharmacy, delirium, cognitive disorders, depression disorders, anxiety disorders, sleep disorders, sarcopenia, frailty, and falls between physicians and nurses (all *P* < 0.05).

**Table 3:** Knowledge toward management procedures for GS and other geriatric-related issues

	Total (*n* = 225)	Range	Physicians (*n* = 129)		Nurses (*n* = 96)		*P* Value
Have you ever diagnosed, treated, or cared about GS? n (%)							0.144
Never/unknown	38(16.9)		19(14.7)		19(19.8)		
Rarely/Occasionally	141(62.7)		78(60.5)		63(65.6)		
Often/Always	46(20.4)		32(24.8)		14(14.6)		
Knowledge of management procedures for GS, scores, median (IQR)							
Comorbidity	3(4.0)	10	4(4.0)		3(3.8)		**< 0.001**
Polypharmacy	4(3.0)	10	5(4.0)		3(4.0)		**0.003**
Swallowing disorders	5(4.0)	10	5(4.0)		5(4.8)		0.854
Malnutrition	5(4.0)	10	5(3.0)		4(4.0)		0.167
Delirium	3(4.0)	10	4(3.5)		3(4.0)		**0.033**
Cognitive disorders	4(3.0)	10	4(4.0)		3(4.0)		**0.006**
Depression disorders	4(3.0)	10	4(4.0)		3(4.0)		**0.005**
Anxiety disorders	4(3.0)	10	4(4.0)		3(4.0)		**0.003**
Sleep disorders	4(3.0)	10	4(4.0)		3(3.0)		**0.012**
Chronic pain	4(3.0)	10	4(4.0)		4(3.0)		0.693
Sarcopenia	2(5.0)	9	3(4.0)		2(3.0)		**0.002**
Frailty	3(4.0)	9	4(3.5)		2(3.0)		**0.004**
Fall	6(4.0)	10	5(4.0)		7(3.8)		**< 0.001**
Urinary incontinence	4(3.5)	10	4(3.0)		4(4.0)		0.383
Multidisciplinary team management for the elderly	4(3.0)	10	4(3.0)		4(4.0)		0.997

*GS* geriatric syndromes; *IQR* interquartile range; Significance difference *P* < 0.05 was shown in bold

### Training needs and barriers to practicing geriatrics

Less than 60% of participants had received training in geriatrics. As shown in Table 4, the top five GS they expressed a desire to systematically learn about were cognitive disorders, malnutrition, sleep disorders, frailty, and sarcopenia, respectively. There was no significant difference between physicians and nurses in their need for training in GS, except for polypharmacy. Further analysis of training needs among physicians and nurses with varying levels of training experience and workplaces were conducted. Results showed that physicians with training experience showed less interest in learning about anxiety disorders (24.7% vs. 44.6%, *P* = 0.017). Nurses with training experience showed a greater interest in learning about delirium compared to those without such experience (33.3% vs. 10.3%, *P* = 0.009). Additionally, physicians working in geriatric departments expressed greater interest in learning about sarcopenia compared to their counterparts in other departments (55.9% vs. 33.7%, *P* = 0.023). On the other hand, nurses in geriatric departments were more interested in learning about delirium (44.4% vs. 15.9%, *P* = 0.003).

**Table 4:** Training needs and barriers to practicing geriatrics

	Total (*n* = 225)	Physicians (*n* = 129)	Nurses (*n* = 96)	*P* Value
Have you received geriatrics training before?, n(%)				0.676
No training experience	95(42.2)	56(43.4)	39(40.6)	
Have training experience	130(57.8)	73(56.6)	57(59.4)	
GS that wish to learn systematically, n(%)				
Polypharmacy	95(42.2)	63(48.8)	32(33.3)	**0.020**
Malnutrition	120(53.3)	70(54.3)	50(52.1)	0.746
Delirium	42(18.7)	19(14.7)	23(24.0)	0.079
Cognitive disorders	148(65.8)	89(69.0)	59(61.5)	0.239
Depression disorders	93(41.3)	50(38.8)	43(44.8)	0.363
Anxiety disorders	79(35.1)	43(33.3)	36(37.5)	0.517
Sleep disorders	118(52.4)	67(51.9)	51(53.1)	0.860
Chronic pain	64(28.4)	39(30.2)	25(26.0)	0.491
Sarcopenia	99(44.0)	51(39.5)	48(50.0)	0.118
Frailty	106(47.1)	62(48.1)	44(45.8)	0.740
Fall	48(21.3)	23(17.8)	25(26.0)	0.137
Urinary incontinence	50(22.2)	25(19.4)	25(26.0)	0.235
The biggest barrier to practicing geriatrics, n(%)				NA*
Unclear direction of discipline development	18(8.0)	11(8.5)	7(7.3)	
Loose specialist talent echelon	24(10.7)	13(10.1)	11(11.5)	
Lack of systematic specialist knowledge and technology	78(34.7)	28(21.7)	50(52.1)	
Limited patient source	5(2.2)	4(3.1)	1(1.0)	
Doctor-patient communication problems	6(2.7)	2(1.6)	4(4.2)	
Caregiving problems	16(7.1)	8(6.2)	8(8.3)	
Medical insurance payment issues	43(19.1)	38(29.5)	5(5.2)	
Insufficient hospital-level support	20(8.9)	14(10.9)	6(6.3)	
Insufficient support from government departments	13(5.8)	10(7.8)	3(3.1)	
Legal security problems	2(0.9)	1(0.8)	1(1.0)	

*GS* geriatric syndromes; *The sample sizes of some options are too small to be analyzed. Significance difference *P* < 0.05 was shown in bold

Physicians identified medical insurance payment issues and a lack of systematic specialist knowledge and technology as the two biggest barriers to practicing geriatrics. Nurses, on the other hand, cited a lack of systematic specialist knowledge and technology as the main barrier, as displayed in Table 4.

## Discussion

Our study revealed that almost two-thirds of geriatric practitioners had encountered CGA in their clinical practices. The percentage of CGA applications in our study is consistent with previous findings. In a survey of 98 Chinese geriatricians from three general hospitals, only 14.4% frequently applied CGA to their older patients, while about 53.6% attempted to apply CGA to their patients [15]. A study conducted in Southwest China found that 75% of the respondents had evaluated at least one item of CGA separately [14]. CGA tools are adjusted to fit actual healthcare settings [13], and several studies have confirmed their cost-effectiveness [19–21]. Another study showed that physicians and nurses recognized specialized geriatric techniques as beneficial for improving patient safety and clinical outcomes [22], which was consistent with our study. Of the detailed CGA evaluation items, malnutrition risk, fall risk, activities of daily living, pain, and cognitive function were the top five CGA evaluation items currently used. Although the percentages of other GS and related geriatric problems such as frailty, sarcopenia, and delirium assessed remain low, it is important to note that these conditions are highly prevalent in older patients and are associated with numerous adverse clinical outcomes [2, 23, 24]. These three conditions are all preventable and treatable if geriatric practitioners identify them early [25–27]. However, limited awareness and knowledge of frailty, sarcopenia, and delirium among healthcare professionals have been already been reported [28–31]. Regarding the knowledge of the management procedures for GS and multidisciplinary team management for the elderly, physicians and nurses demonstrated varying levels of emphasis on GS knowledge, but overall knowledge was at a low to moderate level. This may be due to the limited number of specialist geriatricians and nurses, most of whom come from non-geriatric backgrounds, and have not received formal geriatric education and training (including CGA training) prior to joining the geriatric department. Therefore, geriatric practitioners should be equipped with the necessary awareness and knowledge, including diagnostic strategies and optimal interventions for GS, as well as how to organize a multidisciplinary team.

Physicians and nurses commonly express concern about the barriers to practicing geriatrics, particularly the lack of systematic specialist knowledge and technology. The findings of our study was consistent with those of another study conducted in China [32]. These results may reflect a mismatch between the demand for geriatric specialists and the number of specialists graduating from geriatric continuing medical education programs. To bridge this gap, one solution is to enhance the training of specialized personnel, such as geriatric physicians and geriatric nurses, and provide continuing education to improve their core competence in providing comprehensive services for older adults [32–34]. Although there was little difference in training needs between physicians and nurses, the top five GS for which systematic learning was desired were cognitive disorders, malnutrition, sleep disorders, frailty, and sarcopenia. Geriatric education and continuous training should be designed to address the specific roles of physicians and nurses, as well as the practical knowledge reserve, barriers, and training needs they face. Medical insurance payment issues were identified as a barrier to CGA application. To address this, prioritizing the multi-tiered medical insurance system for older adults with multiple comorbidities at the institutional level is also necessary. Additionally, loose specialist talent echelon, insufficient hospital-level support, and unclear direction in discipline development were identified as significant.

Gladman JR et al. proposed that the implementation of CGA in real-world clinical settings is hindered by a ‘know-do gap’ phenomenon from the perspective of implementation science [35]. They identified seven common domains of the ‘know-do gap’ in implementing CGA, which include guideline factors, professional factors, patient factors, professional interactions, incentives and resources, capacity for organizational change, and social, political, and legal factors [35]. The study showed a big gap between the ideal and the actual proficiency of geriatric practitioners in clinical practices using CGA [15]. The National Health Commission has currently included the construction of geriatric medicine in the scope of monitoring and evaluation of Healthy China Action. The government at all levels has provided unprecedented opportunities for the advancement of geriatric medicine [36]. Efforts should be made to standardize the implementation of CGA in the field of geriatric medicine.

The study had limitations due to selection bias and the sample size. The study did not include geriatric practitioners who did not attend geriatric medicine or geriatric nursing training. The sample size of included physicians and nurses was small, which may limit the generalizability of the findings to all healthcare institutions. To fully reflect the perspectives of geriatric practitioners, large-scale studies with more diverse populations should be conducted to investigate the application of CGA before and after continuing education training in geriatric medicine.

## Conclusion

This study revealed that the rate of CGA application at the individual level, as well as the overall knowledge among geriatric practitioners, was not adequate. Geriatric education and continuous training should be tailored to address the specific roles of physicians and nurses, as well as the practical knowledge reserves, barriers, and training needs they face.

### Supplementary Information

**Supplementary Material 1.**

## Data Availability

The data that support the findings of this study are available from the corresponding author upon reasonable request.
