# Supplementary material for: Application, knowledge and training needs regarding comprehensive geriatric assessment among geriatric practitioners in healthcare institutions: a cross-sectional study
Source: BMC Geriatr. 2024 Apr 18;24:349. doi: 10.1186/s12877-024-04964-9 (PMC11025239; doi:10.1186/s12877-024-04964-9)
Supplement: Supplementary file 1 — Supplementary Material 1. [file 12877_2024_4964_MOESM1_ESM.docx]

Table S1 Questionnaire design

| Q1: Have you had any experience with CGA in your clinical work?  □Never □Rarely □Occasionally □Often □Always □Unknown  If the answer is "Rarely/Occasionally/Often/Always", please specify the CGA specific-evaluation items.  □Comorbidity □Medication □Hearing and vision □Oral and swallowing functions  □Malnutrition risk □Delirium □Cognitive function □Emotion □Sleep quality  □Pain □Activity of daily living □Muscle strength □Frailty □Physical function  □Fall risk □Urinary incontinence |
| --- |
| Q2: Do you believe that CGA is beneficial for clinical diagnosis and treatment?  □Very helpful □helpful □Neutrality □Rather not helpful □Not helpful  □Unknown |
| Q3: Do you believe that CGA is beneficial for clinical care?  □Very helpful □helpful □Neutrality □Rather not helpful □Not helpful  □Unknown |
| Q4: Do you believe that CGA is beneficial for ward safety?  □Very helpful □helpful □Neutrality □Rather not helpful □Not helpful  □Unknown |
| Q5: Is implementing CGA difficult for you?  □Very difficult □Somewhat difficult □Neutral □Somewhat easy  □Very easy □Unknown. |
| Q6: Have you ever diagnosed, treated, or cared about GS?  □Never □Rarely □Occasionally □Often □Always □Unknown |
| Q7: Please score the following given knowledge of management procedures for GS and multidisciplinary team management for the elderly. The degree of knowledge is assessed on a scale score of 0 to 10, with a higher score indicating a greater level of knowledge.  Comorbidity: 0 1 2 3 4 5 6 7 8 9 10  Polypharmacy: 0 1 2 3 4 5 6 7 8 9 10  Swallowing disorders: 0 1 2 3 4 5 6 7 8 9 10  Malnutrition: 0 1 2 3 4 5 6 7 8 9 10  Delirium: 0 1 2 3 4 5 6 7 8 9 10  Cognitive disorders: 0 1 2 3 4 5 6 7 8 9 10  Depression disorders: 0 1 2 3 4 5 6 7 8 9 10  Anxiety disorders: 0 1 2 3 4 5 6 7 8 9 10  Sleep disorders: 0 1 2 3 4 5 6 7 8 9 10  Chronic pain: 0 1 2 3 4 5 6 7 8 9 10  Sarcopenia: 0 1 2 3 4 5 6 7 8 9 10  Frailty: 0 1 2 3 4 5 6 7 8 9 10  Falls: 0 1 2 3 4 5 6 7 8 9 10  Urinary incontinence: 0 1 2 3 4 5 6 7 8 9 10 |
| Q8: Have you received geriatrics training before?  □Systematically trained □Piecemeal trained □Never trained |
| Q9: Which five GS do you wish to learn systematically in this training?  □Polypharmacy □Malnutrition □Delirium □Cognitive disorders  □Depression disorders □Anxiety disorders □Sleep disorders □Chronic pain  □Sarcopenia □Frailty □Fall □Urinary incontinence |
| Q10: What do you believe is the biggest barrier to practicing geriatrics?  □Unclear direction of discipline development  □Loose specialist talent echelon  □Lack of systematic specialist knowledge and technology  □Limited patient source  □Doctor-patient communication problems  □Caregiving problems  □Medical insurance payment issues  □Insufficient hospital-level support |
| □Insufficient support from government departments |
| □Legal security problems |

CGA: comprehensive geriatric assessment; GS: geriatric syndromes.
